# Prenatal and Perinatal Environmental Influences Shaping the Neonatal Immune System: A Focus on Asthma and Allergy Origins

**DOI:** 10.3390/ijerph18083962

**Published:** 2021-04-09

**Authors:** Azahara María García-Serna, Elena Martín-Orozco, Trinidad Hernández-Caselles, Eva Morales

**Affiliations:** 1 Biomedical Research Institute of Murcia (IMIB-Arrixaca), 30120 Murcia, Spain; am.garciaserna@um.es (A.M.G.-S.); emartin@um.es (E.M.-O.); trini@um.es (T.H.-C.); 2 Department of Biochemistry and Molecular Biology B and Immunology, Faculty of Medicine, University of Murcia, 30100 Murcia, Spain; 3 Network of Asthma and Adverse and Allergic Reactions (ARADyAL), 28029 Madrid, Spain; 4 Department of Public Health Sciences, University of Murcia, 30100 Murcia, Spain

**Keywords:** early life, environment, immune system, programming, pregnancy

## Abstract

It is suggested that programming of the immune system starts before birth and is shaped by environmental influences acting during critical windows of susceptibility for human development. Prenatal and perinatal exposure to physiological, biological, physical, or chemical factors can trigger permanent, irreversible changes to the developing immune system, which may be reflected in cord blood of neonates. The aim of this narrative review is to summarize the evidence on the role of the prenatal and perinatal environment, including season of birth, mode of delivery, exposure to common allergens, a farming environment, pet ownership, and exposure to tobacco smoking and pollutants, in shaping the immune cell populations and cytokines at birth in humans. We also discuss how reported disruptions in the immune system at birth might contribute to the development of asthma and related allergic manifestations later in life.

## 1. Introduction

Non-communicable diseases (NCDs) are predominantly chronic diseases that include metabolic and cardiovascular diseases, cancer, autoimmune conditions, neurological disorders, chronic lung disease, asthma and other allergic diseases. Typically, these diseases share common features: early-life exposure to environmental agents, chronic low-grade inflammation, and immune disturbance during development. Growing evidence is showing that environmentally induced disruption of normal immune system development may play a significant role in the current global epidemic of NCDs whose prevalence has dramatically increased worldwide in the last decades [1]. Most of the variability in the immune system is due to environmental factors, rather than genes [2]. Moreover, the enhanced vulnerability of the developing immune system for environmental insults is based on unique immune maturational events that occur during critical windows in early life, which includes the fetus and the first years of life. To this regard, the fetal immune system seems to be shaped and programmed markedly by the *in utero* and the perinatal environment, which can have related adverse health consequences later in life. 

In this review, we summarize the current evidence on the role of the prenatal and perinatal environment in shaping the human immune system at birth, including the influences of physiological, biological, physical, and chemical factors. In particular, we focus on neonatal immune cell populations and cytokines and discuss how reported disruptions at birth might contribute to the development of asthma and related allergic manifestations later in life.

## 2. Immune System Development and Maturation

The immune system is a complex network of cells, proteins, tissues and organs that defends the host against microbes and molecules that are recognized as foreign, and ultimately protects against disease. In humans, there are two principal subsystems: the innate and the adaptive or acquired immune system, each of which comprises cellular and humoral components to perform their functions. 

Leukocytes, the cellular component of the immune system, are divided into myeloid and lymphoid cells. Myeloid leukocytes are the main cellular components of the innate system, which includes granulocyte cells (neutrophils, eosinophils, basophils), monocytes, macrophages, mast cells, and dendritic cells (DC) [3,4]. Innate cells sense, capture and process antigens while they inform nearby cells by presenting antigens to T lymphocytes and secreting cytokines, small proteins involved in signaling. Macrophages are the major cytokine producers, including pro-inflammatory cytokines such as interleukin (IL)-1β, IL-6, IL-12, IL-18 and tumor necrosis factor alpha (TNF-α) [3]. Lymphoid progenitors originate lymphocytes B and T, the main actors in adaptive immune system, and natural killer (NK) cells that are involved in both the innate and the adaptive response. Adaptative immune response produces immunological memory through antibodies released by B cells. T cells can differentiate into T-helper (Th), T-cytotoxic (Tc) or T-regulatory (Treg). Tc and NK cells attack invaders through cytotoxicity; while Th cells promote and control the development of the immune response. Th cells that recognize an antigen can differentiate into different Th types, including Th1, Th2, and Th17 [5]. During fetal development, Th1 response is thought to be inhibited in the maternal-fetal interface and immune response typically switch into a Th2 response to avoid fetal rejection [6,7]. Th2 cells, which are involved in defense against helminths and other extracellular parasites, mainly produce IL-4, IL-5, IL-9 and IL-13, activate mast cells and eosinophils, and stimulate production of IgE [8]. At birth, the bias towards a Th1 response starts in the neonatal system. Th1 cells mainly produce interferon gamma (IFN-γ), activate macrophages (M1 classical activation), NK and Tc cells, and stimulate the production of IgG. Th1-related cells defend the host against intracellular pathogens, including bacteria, parasites, yeast, and viruses [9]. Prenatal and postnatal early life environmental factors can induce an unbalanced Th1/Th2 response that could be responsible for several diseases later in life [6,10]. Thus, a predominant Th1 response is associated with autoimmune diseases [9], while a predominant Th2 response is associated with atopic disease and allergies [8].

Five major maturational periods through immune system development are described as critical windows of vulnerability [11]. Firstly, hematopoietic stem cells (HSCs) first emerge in the aorta–gonad–mesonephros (AGM) region at around 5 weeks of gestational age [4] and, later on, initiation of hematopoiesis occurs in mid first trimester when pluripotent stems cells appear in the yolk sac and in the fetal liver progressively, and myeloid and lymphoid precursors colonize the thymus and the fetal liver [12]. Secondly, between late first trimester and early second trimester of gestation, stem cells migrate and progenitors expand to new and peripheral tissues [12]; lymphocytes precursors seed the thymus where maturation of T cells is rendered [10]. Thirdly, hematopoietic stem cells colonize the bone marrow and the thymus between gestational weeks 15–16 until birth. During the third trimester of gestation, immune cell numbers increase, and DCs initiate their maturation towards a Th1 response that continues during first months of life. Finally, immune competence maturation during the first year of life and the establishment of immunological memory between 1 and 18 years of age constitute the two extrauterine critical windows of immune system development [12].

## 3. Prenatal Environmental Exposures and Susceptibility to Asthma and Allergies

A complex interplay between environmental exposures acting during critical windows of development in early life and genetic susceptibility likely contribute to occurrence of asthma and allergy [13,14]. Identified environmental exposures include physiological, biological, physical, or chemical factors.

Season of birth may influence the occurrence of asthma and allergic manifestations later in life. Children born in autumn have a higher risk of asthma compared with those born in spring [15,16]. Moreover, children born in autumn or winter have a higher risk of food allergy [17,18,19], allergic rhinitis [20] and atopic dermatitis [21] compared to those born in spring or/and summer. 

Mode of delivery has also been identified as an important factor in the occurrence of asthma and allergy. Cesarean section, whose rate has increased in parallel with the prevalence of childhood asthma over the past decades, is related to increased risk of wheeze up to school age [22,23], asthma in childhood [24,25,26,27], and bronchial hyperreactivity in early adulthood [28]. Moreover, cesarean delivery may increase risk of food allergy and atopic dermatitis [29,30,31].

Prenatal and postnatal early life exposure to common allergens [32], mold or/and dampness [33,34], and furry pets’ ownership [32,35,36] are involved in the development of asthma and allergy. Exposure to mold and dampness increases risk of allergic rhinitis, wheezing and atopic eczema in childhood [33,37,38,39]; while a farming environment and ownership of pets could reduce risk of allergic diseases and atopy [40,41,42].

Maternal smoking during gestation is associated with increased risk in the offspring of respiratory infections [43], wheezing [44,45,46], asthma [47,48], and impaired lung function in childhood and later in life [49,50]. Secondhand tobacco smoke during pregnancy could also influence risk of asthma [51]. Indoor environmental pollutants could contribute to development of atopy; performing housing renovation and painting during pregnancy could be implicated in development of asthma [34,38] and atopic eczema [38,52,53] through emissions of volatile organic compounds (VOCs) or dust [54,55]. 

Prenatal exposure to persistent organic pollutants (POPs) such as organochlorine pesticides has been associated with increased risk of wheeze and asthma later in life [56,57,58]. Furthermore, non-persistent organic pollutants have been associated with increased risk of respiratory infections in infancy [59], impaired lung function performance in children [60], and the occurrence of asthma [61,62] and allergies [63,64]. In addition, some studies have shown a higher risk of atopic diseases [65,66] and impaired lung function [67] in childhood in relation to *in utero* exposure to toxic metals. Finally, growing evidence has shown prenatal exposure to air pollutants, primarily NO_2_ and particulate-matter (PM_2.5_ and PM_10_), to be associated with the occurrence of wheezing [68], asthma [32,69,70,71], rhinitis [38,68], eczema [38], and impaired lung function [72] during childhood.

## 4. Prenatal and Perinatal Environmental Influences and Immune System at Birth: Review of the Literature

### 4.1. Search Strategy and Study Selection

A literature search was conducted by two independent reviewers (AMG-S and EM) in MEDLINE (via PubMed) through September 2020. The search strategy used the following keywords: outcome (“leukocytes” OR “lymphocytes” OR “immune system” OR “Th1” OR “Th2” OR “cytokines” OR “IgE”) combined with “cord blood” and with the next keywords for exposures (“season of birth” OR “mode of delivery” OR “cesarean section” OR “vaginal delivery” OR “farming environment” OR “farming” OR “pets” OR “dog” OR “cat” OR “indoor allergens” OR “dust mite” OR “mold” OR “dampness” OR “tobacco smoking” OR “persistent organic pollutants” OR “organochlorine pesticides” OR “volatile organic compounds” OR “metals” OR “arsenic” OR “cadmium” OR “lead” OR “chromium” OR “mercury “ OR “air pollution” OR “nitrogen dioxide” OR “particulate matter”)”. Limits: Human, English. Identification and first screening of the articles were performed using the information available in the title and the abstract. Potentially relevant studies were retrieved in full text and assessed for eligibility; any discrepancies were resolved by discussion between the two independent researchers or by discussion with a third review author.

The selection criteria were: (a) article written in English; (b) original research article based on an epidemiologic study performed in human individuals (abstracts, case reports, comments, and lab-based studies were excluded); (c) outcome assessment included phenotyping of immune system cells (leukocytes, lymphocytes and cellular subsets) or cytokine profile patterns assessed in cord blood of newborns; and d) assessment of season at birth, mode of delivery, farming exposures, pets, common allergens, tobacco smoking, persistent and non-persistent pollutants, toxic metals and outdoor air pollution as exposures during pregnancy or around birth. After screening of retrieved articles, 78 articles met our inclusion criteria, including 49 cohort studies, 28 cross-sectional studies and 1 retrospective study conducted between 1979 and 2020.

### 4.2. Season of Birth

Immune cell distributions [73,74] and cytokines [75,76,77,78,79] in cord blood of neonates have been investigated in relation to season of birth (Table 1). Collinson et al. [73] found higher leukocyte and lymphocyte counts at birth in children born in wet season compared to those born in dry seasons, but no differences were found for lymphocyte subsets. Moreover, compared to neonates born in summer those born in winter also showed higher leukocyte counts, including granulocytes, neutrophils and monocytes, higher number of plasmacytoid dendritic cells and activated Th and NK cells [74]. Tc cell counts increased in neonates born in spring compared with those born in autumn [74]. No associations were found between season of birth and cord blood distributions of B and T cells, nor lymphocytes subsets including Th and Treg cells [74].

Some studies have reported that neonates born during spring or summer time had significantly lower production and response of cytokines, including TNF-α, IFN-γ, IL-5 and IL-10 [75,79]. However, other studies have reported highest induced responses of IL-4, IL-5, IL-13 among neonates born during summer [75,77]. Moreover, neonates born in autumn or winter showed increased cockroach-induced IL-5 [76] and pro-inflammatory cytokine concentrations and response to specific stimuli (IFN-γ, IFN- α, IL-8, TNF-α and IL-12p40) [78].

### 4.3. Mode of Delivery

An increased number of leukocytes in cord blood of neonates is associated with vaginal delivery [80,81,82,83,84,85,86] and longer duration of labor [79] (Table 2). Accordingly, cesarean section is associated with decreased counts of leukocytes [79,87,88,89]. Moreover vaginal delivery is related to increased neutrophils [80,82,83,84,86,90] and monocytes [80,86,91], but decreased lymphocytes at birth [83,84]. In addition, cord blood monocytes of neonates born by cesarean section express lower level of the surface innate antigen receptors toll like receptor 2 (TLR2) and TLR4 [87]. Furthermore, vaginal delivery is associated with increased NK cells [80,84,92,93], a higher expression of CD16 and CD56 surface receptors [92], increased Treg cells [94], and a decreased expression of specific Treg transcription factor FoxP3 [95]. Most of the studies have showed no association between mode of delivery and distributions of B and T cytotoxic cells [80,91,92], or memory and naïve T lymphocytes [91,96] in cord blood. Results on T and Th cells are inconsistent. Although some studies did not find an association between mode of delivery and the distribution of T and Th [80,91] cells; others have reported decreased proportions of T [84,92] and Th cells [92] in neonates born by vaginal delivery.

In addition, mode of delivery may alter cytokine patterns at birth. Compared to those delivered by cesarean section neonates born by vaginal delivery had an enhanced innate response, showing higher IL-1β, IL-6 and IL-8 levels but lower granulocyte colony-stimulating factor (G-CSF) levels [23,86,97,98,99,100]. Results on relationship between TNF-α and mode of delivery are contradictory [98,100]. Moreover, vaginal delivery may induce a Th1-related response (IFN-γ and IL-12) at birth [79,84,86,98,101]. On the other hand, cesarean section was associated with an increased IL-13 response to allergens, PHA and LPS [102], and a decreased Granulocyte-macrophage colony-stimulating factor (GM-CSF) concentrations [89], TLR1-induced pro-inflammatory response (i.e., TNF-α and IL-6) [23], and Th2-related response [78,79] at birth. In addition, IL-12p40 showed decreased response after CpG-stimulation but increased response after viral protein induction [78] in cord blood cells of neonates delivered by cesarean section.

### 4.4. Prenatal Exposure to a Farming Environment, Pets, and Indoor Allergens

Impact of farming-related exposures, pets and indoor allergens on the immune system at birth has been investigated in diverse studies (Table 3). Decreased number of leukocytes [79], increased number and function of Treg cells [103], and increased B-cell activating factor [104] have been found in neonates of farming parents. 

Several studies have investigated cord blood immunoglobulin E (IgE), a key feature in allergic manifestations, in relation to prenatal exposure to common allergens. Total IgE levels decreased in cord blood from newborns whose mothers had been exposed to dogs [105,106] and endotoxins [107] during pregnancy, while increased total IgE levels have been found in cord blood of newborns exposed to cats [106,107] and dust mite [107,108]. Furthermore, specific IgE for seasonal allergens decreased in cord blood of newborns whose mothers were exposed to a farm environment during pregnancy, whereas specific IgE for cow’s milk and food allergens increased in cord blood of newborns whose mothers consumed boiled farm milk during pregnancy [109].

Prenatal farm environment is related to increased proinflammatory cytokines (TFN-α and IL-6) in cord blood of neonates [103,110]. Moreover, ownership of dogs during pregnancy has been associated with decreased TNF-α [111] and ownership of dogs or cats has been related to an increased IL-6 response [112]. Th1-related response through IFN-γ production was increased in newborns exposed to farm animals and among those whose mothers consumed unskimmed farm milk or butter made of farm milk during pregnancy [110]. Although, no associations between prenatal exposure to pets (cats and dogs) and IFN-γ in cord blood of neonates has been reported [113]. Prenatal contact with higher number of animal species was associated with increased dust mite allergen-induced IFN-γ response [110]. However, other studies have reported a decreased IFN-γ response in cord blood of newborns of farmer fathers [75], as well as a decreased LPS-induced IFN-γ response in relation to prenatal exposure to high endotoxin levels [114], and a lower proportions of induced Th lymphocytes producing IFN-γ among newborns prenatally exposed to high house dust mite (Der p 1) [115].

In addition, farm environments could reduce a Th2-related response in newborns. Prenatal exposure to stables was related to a decreased dust mite allergen-induced IL-5 response [103]; and newborns whose fathers were farmers showed a decreased IL-5 and IL-10 induced-response [79]. Moreover, neonates whose mothers consumed yogurt made of farm milk during pregnancy showed decreased IL-5 and IL-10 induced-response in cord blood [110]. However, no associations between prenatal exposure to pets (cats or dogs) and IL-13 were found in cord blood of neonates [113].

### 4.5. Tobacco Smoking

Exposure to maternal tobacco smoking during pregnancy has been associated with a reduction in neutrophils [116,117,118], monocytes [118], lymphocytes [118], and lymphocyte subpopulations including T helper CD4+ cells [119] and Treg cells [120] in cord blood (Table 4). Moreover, some studies have reported lower Th1 related cytokine responses in cord blood of neonates of mother who smoked during pregnancy, including TNF-α [121] and IFN-γ [122]. Neonatal Th2 related cytokine responses to allergen and different stimuli are inconsistent across studies. Reduced responses of Th2-related cytokines (i.e., IL-5 and IL-10) have been found [79,110,121]; however, increased IL-13 response has been reported as well [123]. In addition, reduced levels and induced response to TLR2 and TLR9 pathways of IL-6 have been found in cord blood of neonates whose mothers smoked during pregnancy [121,124].

### 4.6. Persistent and Non-persistent Organic Pollutants

Diverse studies have investigated the effects of organic pollutants on prenatal immune system development [125,126,127,128,129,130,131,132,133,134,135] (Table 5). Neonates born in areas contaminated with high levels of POPs (DDE, HCB and PCBs) showed decreased percentages of Th lymphocytes [125], dendritic cells, resting T cells, suppressor inducer T cells and NK cells [129,130], while increased proportions of B and activated B cells, Tc lymphocytes [130], and memory T cells [129]. To date, no study has examined the association between prenatal exposure to non-persistent organic pollutants and leukocyte and lymphocyte distributions in newborns.

Furthermore, prenatal exposure to organic pollutants has been associated with impaired immunoglobulin contents in cord blood plasma of neonates, including decreased IgM and increased IgG levels [125]. Furthermore, sex may play an important role in toxicity since male newborns prenatally exposed to dioxin-like compounds showed decreased IgE concentrations in cord blood [135]. Moreover, a positive correlation between IgE levels and perfluoroalkyl (PFO) analytes PFOA and PFOS in cord serum has been found among males [132].

Several studies have associated exposure to organic compounds during pregnancy with changes in cord blood cytokines patterns [136,137,138,139]. Pro-inflammatory cytokines, such as IL-1β, IL-6, TNF-α and IL-33/TSLP were found decreased in cord blood of newborns exposed to PCBs, DDE, phthalates and organochlorines [126,133,134], BPA [133,137,138] and VOCs [139], whereas newborns exposed to permethrin showed higher IL-1β levels than those less exposed [131]. Nevertheless, maternal urine phthalates concentrations during first trimester were associated with decreased chemotactic cytokines in cord blood [136]. Studies researching Th1 and Th2 cytokines are scarce [128], but results suggest that prenatal exposure to organic compound impacts Th2/Th1 ratios; increased IL-13 levels in cord blood were associated with increased POPs concentrations in placenta [128] and newborns exposed to VOCs during pregnancy showed increased IL-4 concentrations [139]. Finally, effects of prenatal exposure to VOCs on Th1-related cytokines are unclear [139].

### 4.7. Toxic Metals

Diverse epidemiological studies have examined the effects of prenatal exposure to toxic metals on immune system biomarkers in cord blood of neonates [136,140,141,142,143] (Table 6). Changes in cord blood lymphocytes include a decreased number of activated T naïve cells, decreased percentages of Th, Th memory and activated cells, and increased number of Th2-related T cells in newborns exposed to mercury [125], arsenic [141,142] and cadmium [142]. Moreover, prenatal exposure to arsenic has been associated with an increased T cell proliferation and a decreased Treg suppressor function [141]. Furthermore, Belles-Isles et al. found negative correlations between mercury and cord blood IgM levels but positive correlations between lead and IgG cord blood levels [125].

Effects of prenatal exposure to metals on cytokines patters at birth have also been investigated [134,136,138,140]. Prenatal exposure to metals could impair effector Th response and enhance pro-inflammatory and Th2-related response in newborns. Cord blood levels of pro-inflammatory cytokines (TNFα, IL-8, IL-1β and IFN-γ) showed U-shaped associations in relation to maternal exposure to arsenic in late pregnancy [140]. IL-33/TSLP showed an inverse relationship with maternal exposure to lead in early pregnancy [134], and concentrations of chemotactic cytokines increased in newborns prenatally exposed to several metals [136]. In addition, *in utero* exposure to lead and chromium has been associated with higher IL-13 levels in cord blood [143]. 

### 4.8. Outdoor air Pollutants

Studies examining the relationship between prenatal exposure to outdoor air pollutants and distributions of immune cells in cord blood of neonates [88,144,145,146,147,148] are shown in Table 7. Exposure to higher NO_2_ concentrations 14 days before delivery have been associated with decreased counts of leukocytes, neutrophils and monocytes [88]. Moreover, higher concentrations of NO_2_ derived from traffic during the first trimester of pregnancy were associated with decreased counts of leukocytes, lymphocytes, monocytes and basophils [148]. In addition, increased percentage of B cells has been reported in relation to higher levels of outdoor air pollutants during late pregnancy including PAHs and PM_2.5_ [145,146]. 

Associations between prenatal exposure to outdoor air pollutants and the distribution of NK cells in cord blood are inconsistent. Some studies have showed reduced NK cells in cord blood associated with exposure to PAHs and PM_2.5_ during early gestation (first trimester) [146], and to NO_2_ and PM_2.5_ exposure in early and late gestation [148]. However, other studies have reported increased NK cells in cord blood of neonates in relation to living in a high PM polluted area [144], exposure to PM_10_ during the first trimester of pregnancy [147], and exposure in late pregnancy to PAHs, PM_2.5_, NO_2_ and O_3_ [146,147,148].

As for T lymphocytes, results are also inconsistent. Reduced T cells have been found in newborns living in a high PM polluted area [144], among those exposed to higher PAH and PM_2.5_ in late pregnancy [145,146]. Moreover, several studies have reported a decrease in Th cells in relation to prenatal exposure to outdoor air pollutants, including traffic-related NO_2_ in early pregnancy [148], PAHs and PM_2.5_ in late pregnancy [145,146], and PM_10_ during whole pregnancy [147]. Distribution of Th cell subpopulations in cord blood in relation to prenatal exposure to outdoor air pollutants has been poorly investigated [148]. Garcia-Serna et al. reported increased Th cells in relation to high PM concentration during early pregnancy, increased Th1-related cells in neonates exposed to high traffic-related PM levels during early and late pregnancy, and decreased Th1Th2-related cells in relation to higher levels of PM during pregnancy. Moreover, Treg cells were decreased in relation to high concentrations of traffic-related PM_10_ and NO_2_ 15-days before delivery [148].

Most studies have reported a reduction in Tc cells in newborns associated with prenatal exposure to higher levels of outdoor air pollutants, including short-term exposure in later pregnancy to PAHs and PM_2.5_ [145,146], and long-term exposure to PM_2.5_ derived from traffic [148]. However, Baïz et al. found increased Tc in newborns exposed to high NO_2_ concentrations during the first and second trimester of pregnancy [147]. 

The influence of prenatal exposure to air pollutants on cytokines in cord blood of neonates has been poorly examined [124,149]. Latzin et al. reported increased levels of IL-1β in cord blood serum in relation to short-term exposure to higher PM_10_ concentrations before birth, and decreased levels of IL-10 in relation to higher PM_10_ concentrations in last trimester of pregnancy [124]. In addition, Ashley-Martin et al. found maternal exposure to higher NO_2_ levels to be associated with increased IL-33 and thymic stromal lymphopoietin (TSLP) concentrations in cord blood of neonates, and increased IgE levels among female neonates exposed to higher PM_2.5_ levels during pregnancy [149].

## 5. Discussion

Season of birth, mode of delivery, prenatal exposure to common allergens and chemicals, including tobacco smoking, organic pollutants, metals and outdoor air pollutants, may impair distributions of immune system cells (i.e., leukocytes and lymphocytes), as well as, alter immunoglobulins and cytokine patterns in cord blood of neonates.

Winter birth is associated with increased leukocytes, NK and activated Th cells [74] and a predominant type 1- and type 17-associated immune response with increased concentrations of pro-inflammatory cytokines [78]; whereas spring or summer birth related to a predominant Th2-related cytokine response [75,77]. Findings suggest activation of antimicrobial pathways that might reflect increased maternal exposure to infectious microbes during or before birth, which may serve as a trigger of neonatal immunity during fall, winter, and spring that might prime the immune system in early life. In addition to infections, other season-fluctuating conditions could play a role, including maternal nutrition [150,151], vitamin D status [152], and exposure to common allergens [153,154] and air pollutants [155].

Cord blood of neonates delivered vaginally shows increased leukocytes and some of their subsets (neutrophils, monocytes, NK and Treg cells) [80,81,82,83,84,85,86,90,92,93,94], as well as increased pro-inflammatory cytokines (IL-1β, IL-6 and IL-8) [84,86,97,98,99,100]. Whereas cesarean section is associated with decreased cord blood leukocytes, lymphocytes, and dendritic cells [79,87,88]. Moreover, vaginal delivery seems to be related to a Th1-related response in the newborn characterized by a higher IFN-γ and IL-2 production in response to stimuli and a higher IL-12 response to microbial exposure [84,98,101]. Risk of immune diseases, including asthma, and severity of respiratory infections [25,27,156,157] due to cesarean section likely results from lack of labor-associated stress factors and immune-dysregulation caused by a disturbed gut microbiota immune programming in the offspring at birth [158]. In this sense, redistribution and activation of immune cells under stress of labor may be an adaptive response to enhance defensive mechanisms of the immune system. Furthermore, neonates born by cesarean section are exposed to skin flora instead of birth canal microbes, which alters diversity of the microbes that colonize the gut and thus the activation of the immune response [102,159]. 

A prenatal farming environment is related to lower IgE response to seasonal allergens [109], a higher pro-inflammatory cytokine (IL-6 and TNF-α) [103,110] and Th1-related (IFN-γ) responses [103,110], but decreased Th2-related (IL-5) [79,103] and Treg-related (IL-10) [79,110] responses. Moreover, prenatal ownership of cats is associated with a higher IgE response to allergens (pollen and food) [106,107] and ownership of dogs is associated with a lower IgE response to allergens (pollen) [105,106]. A farm environment, and particularly the exposure to elevated number of animal species, resulting in farm-associated microbial exposures has been proposed to have immunoregulatory properties on the developing innate immune system [160]. According to this model, several key effector mechanisms of allergic inflammation are inhibited by the immunoregulatory properties of farm-associated microbial exposures.

Maternal smoking during pregnancy impacts both innate and adaptive immunity of neonates, which could attenuate or exacerbate pathogenic immune responses against infections in early life. Reduced number of leukocytes, lymphocytes, including T helper CD4+, and detrimental IFN-γ response may impair infant defense system against respiratory viral infections in early life, which likely contributes to increased incidence and severity of these infections [160], and thereby to higher asthma susceptibility in childhood [43,47,48]. Moreover, an imbalance of Treg cells is associated with airway inflammation in asthma and allergic rhinitis [161].

Although more evidence is warranted, current evidence suggests that prenatal exposure to POPs might contribute to a failed innate immune responses (i.e., decreased dendritic, T and NK cells), and induce lymphocyte activation (increased B cells and IgG production) and a Th2-related response in neonates [125,129,130]. Moreover, prenatal exposure to both persistent and non-persistent organic pollutants is related to a decreased pro-inflammatory cytokine response (TNF-α, IL-6 and IL-33/TSLP) in neonates [126,133,134,137,138]. Overall, reported disturbances in the immune system of neonates could predispose to a higher susceptibility to respiratory infections during the early postnatal period and thus to increased risk of wheeze and asthma in childhood [56,57,58,59].

Prenatal exposure to metals may impair the innate immune response at birth through changes in T, Treg and Th memory cells [125,141,142], which could contribute to a higher risk of respiratory infections in early life [162,163,164]. In addition, prenatal exposure to metals seem to increase a Th2-related cytokine response in neonates [140,143], which might contribute to development of atopic manifestations later in life [66].

*In utero* exposure to outdoor air pollutants impairs leukocyte and lymphocyte distributions in neonates; early and late gestation seem to be potential developmental windows of higher susceptibility [88,144,145,146,147,148]. Current evidence suggests that prenatal exposure to outdoor air pollution could disbalance Th subsets in neonates reflecting an inflammatory state, which could predispose to allergic immune responses later in life. Reduced Tc cells at birth in relation to *in utero* exposure to outdoor air pollutants may result into a higher risk of respiratory tract infections in childhood given the role of Tc cells in the defense against virus infections [165]. Limited evidence on cytokine profiles in neonates suggests a higher production of pro-inflammatory cytokines (IL-1β and IL-33) in relation to exposure to higher concentrations of NO_2_ and PM [124,149].

This review has some limitations. Studies with small sample size were included in the current review. However, our aim was to summarize all the evidence to date on the impact of prenatal and perinatal environmental influences on immune system at birth. Publication bias cannot be discarded, because studies with no significant findings are less likely to be published. Only articles published in English were included. Finally, heterogeneity between studies in exposure assessment, as well as the small number of studies for any given exposure–outcome relationship, currently make the combination of studies for meta-analysis impossible, but this review summarizes the current evidence and may guide future studies.

## 6. Conclusions

The prenatal and perinatal periods seem to represent crucial biological windows of opportunity for environmental influences to shape the neonatal immune system. Identified disturbances in immune cell populations and cytokines at birth could lead to a higher susceptibility to respiratory infections, asthma and allergic manifestations later in life. Although some associations and mechanisms warrant further investigation, overall, promoting a proper immune system development during early life should be recognized as a major element in the public health agenda to prevent NCDs, especially asthma and allergic manifestations.

## Figures and Tables

**Table 1:** Season of birth and changes in the immune system of neonates (authors ordered by year and then alphabetically).

Author, Year [Ref.]	Location Study Design N	Outcomes Assessed		Season of Birth	Statistically Significant Main Findings
		Immune Cells Frequency	Cytokine Patterns/Ig		
Lehmann et al. 2002 [75]	Germany Cohort N = 158		Cytokine response	Summer	↓ induced-TNF-α (T cells) ↓ induced-IFN-γ (T cells) ↑ induced-IL-4 (T cells)
Sullivan Dillie et al. 2008 [77]	USA Cohort N = 272		Cytokine response	Spring or summer	↑ PHA-induced IL-5 ↑ PMA-induced IL-5 (summer) ↑ Staphylococcus aureus-induced IL-5 ↑ PHA-induced IL-13 ↑ PMA-induced IL-13
				Autumn or winter	↑ CBMC proliferation ↓ Staphylococcus aureus-induced IL-13
Collison et al. 2008 [73]	Gambia Cohort N = 138	Lymphocytes (counts)		Wet season	↑ leukocytes ↑ lymphocytes
Lendor et al. 2008 [76]	USA Cohort N = 350		Cytokine response IgE	Winter	↑ cockroach-induced IL-5
Gold et al. 2009 [78]	USA Cohort N = 558		Cytokine production	Autumn or winter	↑ IFN-γ, IL-8 and TNF-α ↑ IL-12p40
					↑ LPS-induced IFN-α ↑ PG-induced IFN-α ↑ CpG-induced IL-8, TNF-α, and IL-10 ↑ cockroach-induced IFN-γ ↑ dust mite-induced IFN-γ ↑ tetanus toxoid-induced IFN-γ
				Spring/ summer	↑ CpG-induced IFN-α
Keski-Nisula et al. 2010 [79]	Finland Cohort N = 423	Leukocytes (counts)	Cytokine response	Spring	↓ P/I-induced IL-5, IL-10 and IFN-γ
Thysen et al. 2016 [74]	Denmark Cohort N = 84	Leukocytes Lymphocytes (counts)		Winter	↑ Leukocytes, granulocytes, neutrophils and monocytes ↑ Plasmacytoid dendritic cells ↑ CD56 bright NK cells ↑ Activated Th cells
				Spring	↑ Tc cells

CBMC: cord blood mononuclear cell; CD: cluster of differentiation; CpG: Immunostimulatory CpG oligonucleotides; IFN: interferon; IL: interleukin; LPS: Lipopolysaccharide; NK: natural killer; PHA: Phytohemagglutinin; PMA: phorbol 12-myristate 13-acetate; P/I: phorbol ester and ionomycin PG: Peptidoglycan; Tc: T cytotoxic cells; Th: T helper cell; TNF: tumor necrosis factor.

**Table 2:** Mode of delivery and changes in the immune system of neonates (authors ordered by year and then alphabetically).

Author, Year [Ref.]	Location Study Design N	Outcomes Assessed		Mode of Delivery	Statistically Significant Main Findings
		Immune Cells Frequency	Cytokine Patterns/Ig		
Samelson et al. 1992 [92]	USA Cross-sectional N = 12	Lymphocytes (%)		Vaginal delivery	↓ T and Th cells ↑ NK cells
Thilaganathan et al. 1994 [80]	UK Cross-sectional N = 40	Leukocytes Lymphocytes (counts)		Vaginal delivery	↑ Leukocytes, neutrophils, monocytes and NK cells
Nikischin et al. 1997 [81]	Germany Cross-sectional N = 121	Leukocytes (counts)		Vaginal delivery	↑ Leukocytes
Chirico et al. 1999 [82]	Italy Cross-sectional N = 203	Leukocytes (counts)		Vaginal delivery	↑ Leukocytes and neutrophils
Grönlund et al. 1999 [83]	Finland Cross-sectional N = 64	Leukocytes (counts and %)		Vaginal delivery	↑ Leukocytes and neutrophils ↓ Lymphocytes
Kotiranta-Ainamo et al. 1999 [91]	Finland Cross-sectional N = 50	Lymphocytes Macrophages (%)		Cesarean section	↓ CD14+ cells
Steinborn et al. 1999 [97]	Germany Cross-sectional N = 84		Cytokine production	Vaginal delivery	↑ IL-6 in myelomonocytic cord blood cells
Brown et al. 2003 [101]	USA Cross-sectional N = 16		Cytokine response	Vaginal delivery	↑ ConA-induced IFN-γ ↑ LPS-induced IFN-γ and IL-12 ↑ PHA-induced IFN-γ
Gessler et al. 2003 [90]	Switzerland Cross-sectional N = 30	Neutrophils (counts)		Vaginal delivery	↑ Neutrophils ↑ E.Coli-induced phagocytic respiratory burst
Thornton et al. 2003 [96]	UK Cross-sectional N = 27	Leukocytes (%) Lymphocytes (%)	Cytokine response	Cesarean section	↑ CD62L+ Th cells
Malamitsi-Puchner et al. 2005 [98]	Greece Cohort N = 78		Cytokine production	Vaginal delivery	↑ IFN-γ and IL-1β levels ↑ IL-6 and TNF-α levels
Ly et al. 2006 [102]	USA Cohort N = 37		Cytokine response	Cesarean section	↑ IFN-γ baseline secretion ↑ PHA-induced IFN-γ response ↑ cat dander allergen-induced IFN-γ response ↑ induced IL-13 response
Yektaei-Karin et al. 2007 [86]	Sweden Cross-sectional N = 168	Leukocytes	Cytokine production	Vaginal delivery (compared to C-section)	↑ IL-8 ↑ Leukocytes, neutrophils and monocytes
				Assisted delivery (compared to C-section)	↑ IFN-γ ↑ IL-8 ↑ Leukocytes, neutrophils, lymphocytes and monocytes
Gold et al. 2009 [78]	USA Cohort N = 609		Cytokine response	Cesarean section	↓ LPS-induced IL-8 response ↓ PG-induced IL-8 response ↓ CpG-induced IL-12p40 response ↑ Respiratory syncytial virus-induced IL-12p40 response ↓ PHA-induced IL-13 response
Shen et al. 2009 [87]	Taiwan Cross-sectional N = 62	Leukocytes (counts)		Cesarean section	↓ Leukocytes, neutrophils, lymphocytes and monocytes ↓ TLR2 and TLR4 surface expression on monocytes
Keski-Nisula et al. 2010 [79]	Finland Cohort N = 423	Leukocytes (counts)	Cytokine response	Cesarean section	↓ Leukocytes ↓ induced IL-5 response
				Long labour	↑ Leukocytes ↑ induced IFN-α and IL-5 response
				Prostaglandin induction	↓ induced IFN-γ response
Steinborn et al. 2010 [95]	Germany Cross-sectional N = 96	Lymphocytes		Vaginal delivery	↓ FoxP3 expression in Tregs cells
Bili et al. 2011 [93]	Greece Cross-sectional N = 81	Lymphocytes (%)		Vaginal delivery	↑ NK cells
Yildiran et al. 2011 [94]	Turkey Cross-sectional N = 39	Lymphocytes (counts)		Vaginal delivery	↑ Treg cells
Almanzar et al. 2015 [84]	Austria Cohort N = 120	Lymphocytes (counts and %)	Cytokine production	Vaginal delivery	↑ Leukocytes (counts) ↑ Neutrophils and NK cells (%) ↓ Lymphocytes and T cells (%) ↑ IFN-γ, IL-2 and IL-8
Birle et al. 2015 [85]	Germany Cohort N = 66	Leukocytes (counts)		Vaginal delivery	↑ Leukocytes
Treviño-Garza et al. 2016 [99]	Mexico Cross-sectional N = 125		Cytokine production	Vaginal delivery	↑ IL-6
Liao et al. 2017 [23]	Taiwan Cohort N = 487		Cytokine response	Cesarean section	↓ TLR1-stimulated TNF-α and IL-6
Lurà et al. 2018 [88]	Switzerland Cohort N = 289	Leukocytes (counts)		Cesarean section	↓ Leukocytes, basophilic granulocytes ↓ Plasmacytoid dendritic cells
Werlang et al. 2018 [89]	Brazil Cross-sectional N = 64	Leukocytes (counts)	Cytokine production	Cesarean section	↓ Leukocytes ↓ GM-CSF
Nandanan et al. 2019 [100]	Singapore Cohort N = 98		Cytokine production	Vaginal delivery	↑ IL-6 and IL-8 ↓ TNF-α and G-CSF levels

CD: cluster of differentiation; ConA: Concanavalin A; G-CSF: Granulocyte-colony stimulating Factor; CpG: Immunostimulatory CpG oligonucleotides; E.Coli: Escherichia coli; GM-CSF: Granulocyte-macrophage colony-stimulating factor; IL: interleukin; IFN: interferon; LPS: Lipopolysaccharide; NK: natural killer; PHA: P Phytohemagglutinin; PG: Peptidoglycan; Th: T helper cell; Treg: T regulatory cells; TLR: Toll-like receptor; TNF: tumor necrosis factor.

**Table 3:** Prenatal exposure to a farming environment, pets and common allergens and changes in the immune system of neonates (authors ordered by exposure, year and then alphabetically).

Author, Year [Ref]	Location Study Design N	Outcomes Assessed		Factor Assessed	Statistically Significant Main Findings
		Immune Cells Frequency	Cytokine Patterns/Ig		
**Farming environment**					
Ege et al. 2008 [109]	5 European countries (rural areas) Cohort N = 922		Specific IgE levels (food and common inhalants)	Maternal farm exposures	↓ grass pollen-specific IgE ↓ seasonal allergens -specific IgE ↑ cow’s milk-specific IgE ↑ food allergens -specific IgE
				Consumption of boiled farm milk	↑ cow’s milk-specific IgE ↑ food allergens-specific IgE
				Maternal exposure to animal sheds, contact to cattle, removing dung, cleaning the henhouse, handling silage and hay during pregnancy	↓ seasonal allergens-specific IgE
Schaub et al. 2009 [103]	Germany Cohort N = 82	Treg cells (counts)	Cytokine responses	Farming mothers	↑ Treg number and function ↓ Der p 1 plus PG-induced IL-5 ↑ Der p 1-induced IL-6 ↑ Der p 1 plus PG-induced IL-6
			Cytokine responses	Stables	↑ PG-induced CD4+CD25+ T cells ↓ Der p 1 plus PG-induced IL-5
			Cytokine responses	Number of animal species ≥2	↑ IL-10 ↑ Der p 1-induced IFN-γ ↑ PG-induced IFN-γ ↓ Der p 1 plus PG-induced IL-5
Keski-Nisula et al. 2010 [79]	Finland Cohort N = 423	Leukocytes (counts)	Cytokine responses	Father as farmer	↓ Leukocytes ↓ induced IFN-γ, IL-5 and IL-10
Pfefferle et al. 2010 [110]	5 European countries (rural areas) Cohort N = 625		Cytokine response	Maternal farm exposures	↑ induced IFN-γ
				House spent in barn	↑ induced TNF-α
				Contact with farm animal species Consumption of unskimmed farm milk Consumption of butter made of farm milk	↑ induced TNF-α and IFN-γ
				Consumption of yogurt made of farm milk	↓ induced IL-5 and IL-10
				Consumption of cheese made of farm milk	↑ induced IL-5
Lundell et al. 2015 [104]	Sweden Cohort N = 65		Cytokine production	Farming mothers	↑ B-cell activating factor (BAFF)
**Indoor allergens**					
Heinrich et al. 2002 [107]	Germany Cohort N = 1332		IgE	Endotoxin	U-shaped association with IgE ↓ IgE (medium exposure to endotoxin)
				Dust mite (Der p 1)	↑ IgE (medium exposure to mite allergen)
Roponen et al. 2005 [112]	Finland Cross-sectional N = 29		Cytokine response	Endotoxin (in settled dust)	↑ induced-IL-6
Hagendorens et al. 2004 [115]	Belgium Cohort N = 22	Lymphocytes (%)	T cell cytokine production	Dust mite (Der p 1) during second trimester	↓ IFN-γ producing induced Th lymphocytes
Peters et al. 2009 [108]	USA Cohort N = 301		IgE	Dust mite (Der f 1 + Der p 1)	↑ Dust mite-specific IgE
				Cockroach (Bla g 1 + Bla g 2)	↓ cockroach-specific IgE (indirectly)
Lappalainen et al. 2012 [114]	Finland Cohort N = 228		Cytokine response	Staphylococcal enterotoxin B	↓ LPS-induced IFN-γ
				Mycobacterium spp.	↓ LPS-induced IL-8
				Combined chemical markers	↑induced TNF-α
**Pets**					
Heinrich et al. 2002 [107]	Germany Cohort N = 1332		IgE	Cat-allergen exposure	↑ IgE
Roponen et al. 2005 [112]	Finland Cross-sectional N = 29		Cytokine response	Cat/Dog	↑ induced IL-6
Aichbhaumik et al. 2008 [105]	USA Cohort N = 1049		IgE	Cats Dogs	↓ IgE
Sybilski et al. 2009 [106]	Poland Retrospective study N = 173		IgE	Cats	↑ grass and grain pollen-specific IgE ↑ food-specific IgE
				Dogs	↓ grass and grain pollen-specific IgE
Lappalainen et al. 2010 [111]	Finland Cohort N = 228		Cytokine response	Dogs	↓ induced TNF-α
Uzuner et al. 2013 [113]	Turkey Cross-sectional N = 62		Cytokine production	Pets	NS

Der: Dermatophagoides pteronyssinus; IFN: interferon; Ig: Immunoglobulin; IL: interleukin; LPS: lipopolysaccharide; NS: no significant results PG: peptidoglycan; Treg: T regulatory cells; TNF: tumor necrosis factor.

**Table 4:** Prenatal exposure to tobacco smoking and changes in the immune system of neonates (authors ordered by year and then alphabetically).

Author, Year [Ref.]	Location Study Design N	Outcomes Assessed		Tobacco Smoking	Statistically Significant Main Findings
		Immune Cells Frequency	Cytokine Patterns/Ig		
Harrison 1979 [116]	- Cross-sectional N = 257	Leukocytes		Maternal smoking	↓ neutrophils
Mercelina-Roumans et al. 1996 [117]	The Netherlands Cohort N = 142	WBC Leukocytes Reticulocytes		Maternal smoking	↓ neutrophils
Noakes et al. 2003 [123]	Australia Cross-sectional N = 57		Cytokine response	Maternal smoking	↑ IL-13 response to HDM and OVA
Noakes et al. 2006 [121]	Australia Cohort N = 122		Cytokine response	Maternal smoking	↓ TNF-α responses via TLR2, 3 and 4 ↓ IL-6 responses via TLR2 and 9 ↓ IL-10 via TLR2
Pachlopnik Schmid et al. 2007 [118]	Switzerland Cohort N = 97	Leukocytes Lymphocytes		Maternal smoking	↓ neutrophils ↓ lymphocytes ↓ monocytes ↓ myeloid precursor dendritic cells
Karwowska et al. 2008 [119]	Poland Cohort N = 79	Lymphocytes	IgE	Maternal smoking	↓ T helper (CD4+) cells ↑ IgE
Keski-Nisula et al. 2010 [79]	Finland Cohort N = 423		Cytokine response	Maternal smoking	↓ induced IL-5 response
Pfefferle et al. 2010 [110]	5 European countries (rural areas) Cohort N = 625		Cytokine response	Maternal smoking	↓ induced-IL-5 levels
Latzin et al. 2011 [124]	Switzerland Cohort N = 265		Cytokine production	Maternal smoking	↓ IL-6 levels
Hinz et al. 2012 [120]	Germany Cohort N = 346	Treg cells (counts)		Maternal smoking	↓ Treg cells
Sevgican et al. 2012 [122]	USA Cohort N = 277		Cytokine response		↓ mitogen-stimulated IFN-γ

CD: cluster of differentiation; HDM: house dust mite; Ig: Immunoglobulin; IL: interleukin; OVA: ovalbumin; TNF: tumor necrosis factor; Treg: T regulatory cells; TLR: Toll-like receptor; WBC: white blood cells.

**Table 5:** Prenatal exposure to environmental organic pollutants and changes in the immune system of neonates (authors ordered by year and then alphabetically).

Author, Year [Ref.]	Location Study Design N	Outcomes Assessed		Pollutant	Statistically Significant Main Findings
		Immune Cells Frequency	Cytokine Patterns/Ig		
Belles-Isles et al. 2002 [125]	Canada Cross-sectional N = 108	Lymphocytes (%)	IgM IgG	Fishing family	↓ Th naïve cells ↓ IgM and ↑ IgG
				PCBs and p,p′-DDE (cord blood)	↑ IgG
Lehmann et al. 2002 [139]	Germany Cohort N = 85		T cell cytokine response	VOCs	↓ induced IFN-γ response (tetrachloroethylene) ↑ induced IFN-γ response (ethylbenzene and m,p-xylene) ↑ induced IL-2 response (hexane, dodecanem and tridecane) ↓ induced IL-2 response (trichloroethylene and tetrachloroethylene) ↑induced IL-4 response (methylcyclopentane, m,p-xylene and naphthalene) ↓ TNF-α response (methylcyclopentane¸ cyclohexane and tetrachoroethylene)
Bilrha et al. 2003 [126]	Canada Cross-sectional N = 112	Lymphocytes	Cytokine response IgG	Fishing group	↓ PHA-induced TNF-α
				Chlorinated pesticides ΣPCBs (cord blood)	Negative correlations between TNF-α and PCBs, p,p′-DDE, and HCB
Noakes et al. 2006 [121]	Australia Cohort N = 31		Cytokine response	Organochlorines PCBs	NS
Brooks et al. 2007 [128]	USA Cohort N = 19		Cytokine production	Placental p,p′-DDE	↑ IL-13 levels ↑ IL-4/IFN-γ and ↑ IL-13/IFN-γ ratio
Horváthová et al. 2011 [129]	Slovakia Cross-sectional N = 362	Leukocytes (%) Lymphocytes (%)		Living in a region with high levels of PCB contamination	↓ Dendritic-like cells ↓ Myeloid dendritic cells ↑ Memory T cells ↓ Naive/resting T cells ↓ Suppressor inducer T-cells CD4+CD62L+ ↓ Truly naive helper/inducer T-cells CD4+CD62L+CD45RA+ ↑Terminally differentiated effector memory T-cells CD4+CD62L−CD45RA+
Horváthová et al. 2011 [130]	Slovakia Cross-sectional N = 362	Leukocytes Lymphocytes		Living in a region with high levels of PCB contamination	↑ B cells (%) ↑ Activated B cells (%) ↑ Tc cells (%) ↓ NK cells (%)
Neta et al. 2011 [131]	USA Cross-sectional N = 272		Cytokine production	Cis- and trans-Permethrin (Cord serum)	↑ IL-1β levels ↓ Anti-inflammatory response (+IL-10-IL-12p70)
				Oxychlordane (Cord serum)	↓ IL-1β levels
				Trans-nonachlor Piperonyl butoxide (Cord serum)	NS
Wang et al. 2011 [132]	Taiwan Cohort N = 244		IgE	PFOA and PFOS (Cord serum)	↑ IgE (boys)
				PFNA (Cord serum)	NS
Ashley-Martin et al. 2015 [133]	Canada Cohort N = 1258		TSLP IL-33 IgE	Phthalates in maternal urine at first trimester	Inverse non-linear association between maternal urinary MCPP and IL-33/TSLP levels Inverse non-linear association between maternal urinary MCPP and IgE levels
				BPA, maternal urine 1st trimester	Inverse non-linear association between maternal urinary BPA and IL-33/TSLP levels
Ashley-Martin et al. 2015 [134]	Canada Cohort N = 1258		TSLP IL-33 IgE	Organochlorines maternal plasma at 1st trimester	↓ IL-33/TSLP (DDE)
				Organophosphates in maternal urine at 1st trimester	↓ IL-33/TSLP (DEP + DETP)
				PCB, maternal plasma 1st trimester	↓ IL-33/TSLP (PCB 118)
Liao et al. 2016 [137]	Taiwan Cohort N = 275		Cytokine response	BPA	↓ TLR3- and TLR4-stimulated TNF-α response ↓ TLR7-8-stimulated IL-6 response
Huang et al. 2017 [138]	Taiwan Cohort N = 241		Cytokine production	BPA	↓ TNF-α levels
Miyashita et al. 2018 [135]	Japan Cohort N = 268		IgE	Dioxin-like compounds, maternal blood, 2nd and 3rd trimesters	↓ IgE (boys)
Kelley et al. 2019 [136]	USA Cohort N = 56		Cytokine production	12 phthalates in maternal urine, 1st trimester	↑ MIP-α (MnBP) ↑ MCP-3 (MEHP)

BPA: bisphenol A; DEP: Diethylphosphate; p,p′-DDE: p,p′-Dichlorodiphenyldichloroethylene; DETP: Diethylthiophosphate; HCB: hexachlorobenzene; IFN: interferon; Ig: Immunoglobulin; IL: interleukin; MCPP: Mono-3-carboxyypropylphthalate; MIP: Macrophage Inflammatory Proteins; MCP: monocyte chemotactic protein; MnBP: mono n-butyl phthalate; MEHP: mono (2-ethylhexyl) phthalate; NK: Natural Killer cells; NS: no statistically significant results; PAH: polycyclic aromatic hydrocarbons; PCB: Polychlorinated biphenyls; PFOA: Perfluorooctanoic Acid; PFOS: perfluorooctane sulfonate; PFNA: perfluorononanoic acid; PHA: Phytohemagglutinin; PM: particle-matter; Tc: T cytotoxic cells; Th: T helper cells; TLR: toll-like receptor; TNF: tumor necrosis factor; TSLP: thymic stromal lymphopoietin; VOCs: volatile organic compounds.

**Table 6:** Prenatal exposure to metals and metalloids and changes in the immune system in cord blood of neonates (authors ordered by year and then alphabetically).

Author, Year [Ref.]	Location Study Design N	Outcomes Assessed		Metal or Metalloid Assessed	Statistically Significant Main Findings
		Immune Cells Frequency	Cytokine Patterns/Ig		
Belles-Isles et al. 2002 [125]	Canada Cross-sectional N = 108	Lymphocytes (%)	IgM IgG	Hg	↓ Th naïve cells ↓ IgM
				Pb	↑ IgG
Ahmed et al. 2011 [140]	Bangladesh Cohort N = 130		Cytokine production	As	U-shaped association with proinflammatory cytokines IL-1β, IL-8, IFN-γ, TNF-α
Nadeau et al. 2014 [141]	USA Cohort N = 116	Lymphocytes (counts)		As	↓ naïve activated T cells (CD69+) ↑ Th2 cells (CD69− CD294+) ↑ T cell proliferation ↓ Treg suppressor function
Ashley-Martin et al. 2015 [134]	Canada Cohort N = 1258		TSLP IL-33 IgE	Pb in maternal blood at 1st trimester	↓ IL-33/TSLP ODDS
Nygaard et al. 2017 [142]	USA Cohort N = 63	T-lymphocytes		As in toenails (8 wks. of post-partum)	↓ Th memory cells ↓ activated Th memory cells (boys)
				Cd in toenails within 8 weeks of post-partum	↓ Th memory cells (girls) ↓ activated Th memory cells
Kelley et al. 2019 [136]	USA Cohort N = 56		Cytokine production	As, Ba, Be, Cd, Cr, Cu, Hg, Mn, Mo, Ni, Pb, Se, Sn, Tl, U, W, Zn	↑ MIP-α (Mo, Cd, Zn) ↑ MCP-3 (Mo, Ni, Zn)
Kim et al. 2019 [143]	Korea Cohort N = 331		Cytokine production	Pb, Cr	↑ IL-13

CD: cluster of differentiation; IFN: interferon; Ig: Immunoglobulin; IL: interleukin; MIP: Macrophage Inflammatory Proteins; MCP: monocyte chemotactic protein; Th: T helper cells; Th2: T helper type 2 cells; TNF: tumor necrosis factor; Treg: T regulatory cells; TSLP: thymic stromal lymphopoietin; wks.: weeks.

**Table 7:** Prenatal exposure to outdoor air pollutants and changes in the immune system in cord blood of neonates (authors ordered by year and then alphabetically).

Author, Year [Ref.]	Location Study Design N	Outcomes Assessed		Pollutant	Statistically Significant Main Findings
		Immune Cells Frequency	Cytokine Patterns/Ig		
Hertz-Picciotto et al. 2002 [144]	Czech Republic Cross-sectional N = 518	Lymphocytes (%)		Living in a high PM polluted area	↑ NK cells ↓ T-lymphocytes
Hertz-Picciotto et al. 2005 [145]	Czech Republic Cross-sectional N = 1397	Lymphocytes (%)		PAH 14 days before birth	↓ T-lymphocytes ↓ Tc and Th cells ↑ B-lymphocytes
				PM_2.5_ 14 days before birth	↓ T-lymphocytes and Th cells ↑ B-lymphocytes
Herr et al. 2010 [146]	Czech Republic Cohort N = 1397	Lymphocytes (%)		PAHs PM_2.5_	*1st Trimester:* ↑ T-lymphocytes and Th cells ↓ NK cells *3rd Trimester:* ↑ B-lymphocytes and NK cells ↓ T-lymphocytes and Th cells ↓ Tc cells (9th month)
Baïz et al. 2011 [147]	France Cohort N = 370	Lymphocytes (%)		NO_2_	*1st and 2nd Trimester:* ↑ Tc cells (CD8+) *3rd Trimester:* ↑ NK cells
				PM_10_	*1st Trimester:* ↓ T cells ↑ NK cells *1st, 2nd and 3rd Trimester:* ↓ CD25+ Th-cells
				Benzene	↓ CD4+CD25+ T cells
Latzin et al. 2011 [124]	Switzerland Cohort N = 265		Cytokine production	PM_10_ last 3 days of pregnancy	↓ IL-10
				PM_10_ last 3 mo. of pregnancy	↑ IL-1ß
Ashley-Martin et al. 2016 [149]	Canada Cohort N = 1081		IL-33 and TSLP levels	NO_2_	↑ IL-33 and TSLP (girls) ↑ IL-33/TSLP (3rd trimester, girls)
				Whole pregnancy and 1st trimester PM_2.5_	↑ IgE (girls)
Lurà et al. 2018 [88]	Switzerland Cohort N = 295	Leukocytes Lymphocytes (counts)		NO_2_ 14 days before delivery	↓ leukocytes, neutrophils, and monocytes
García-Serna et al. 2020 [148]	Spain Cohort N = 190	Leukocytes Lymphocytes (counts)		Traffic-related NO_2_	*1st Trimester:* ↓ leukocytes, lymphocytes, monocytes and basophils ↓ NK and T cells ↓ Th, Th2 and Th17 cells ↓ Tc cells *2nd Trimester:* ↑ T and Th cells *3rd Trimester:* ↓ Th1Th2-related cells *15 days before delivery:* ↓ NK and Th1Th2 cells *Whole pregnancy:* ↓ NK cells
				Traffic-related PM_2.5_	*1st Trimester:* ↑ Th, Th1 and Treg cells *2nd Trimester:* ↓ Th1 and Th1Th2 cells *3rd Trimester:* ↓ NK cells ↑ Th17 cells *15 days before delivery:* ↑ Th1 cells *Whole pregnancy:* ↓ Tc and Th1Th2 cells
				Traffic-related PM_10_	*1st Trimester:* ↑ T, Th, Th1 and Th1Th2 cells *2nd Trimester:* ↓ Th1, Th17, Th2 and Th1Th2 *3rd Trimester:* ↑ Th1 *15 days before delivery:* ↓ Treg cells *Whole pregnancy:* ↓ Th1Th2 cells
				Traffic-related O_3_	*1st Trimester:* ↑ Th1 cells *2nd Trimester:* ↓ Th1Th2 cells *15 days before delivery:* ↑ NK cells *Whole pregnancy:* ↓ Th17 cells

IL: interleukin; NK: natural killer; PAH: polycyclic aromatic hydrocarbons; PM: particulate matter; Tc: T cytotoxic cells; Th: T helper cells; Th1: T helper type 1 cells; Th2: T helper type 2 cells; Th17: T helper type 17 cells; Treg: T regulatory cells; TSLP: thymic stromal lymphopoietin.

## Data Availability

Not applicable.
